# Supplementary material for: Programmed mitophagy at the oocyte-to-zygote transition promotes species immortality
Source: Res Sq. 2025 Apr 9:rs.3.rs-6330979. Preprint. [Version 1] doi: 10.21203/rs.3.rs-6330979/v1 (PMC12036463; doi:10.21203/rs.3.rs-6330979/v1)
Supplement: 1 [file NIHPPRS6330979V1-supplement-1.pdf]

Supplementary information

Supplementary Table 1. *C. elegans* strains used in the study. Strain name, genotype description, and resource used.

***C. elegans* strains used in this study**		
**Strain**	**Description**	**Resource**
N2	Wild-type	Caenorhabditis Genetics Center (CGC)
NK2747	*qy132 [tomm-20::mNG]* V	This study
NK2845	*qy174 [nduv-2::mNG]* V	This study
EU2917	*or1941[drp-1::GFP]* IV	Caenorhabditis Genetics Center (CGC)
DCR4521	*ola274[atg-9::GFP]* V	Caenorhabditis Genetics Center (CGC)
WEH722	*Si[pVIG57: Pmex-5::mCherry::LGG-2::tbb-2 3'UTR, C.b. unc-119(+)]* II;<br>*unc-119(ed3)*; *ruIs32[pAZ132: pie1::GFP::H2B, unc-119(+)]* III	S Kolli et al., 2024
PHX5270	*syb5270[ctns-1::wrmScarlet]* II	Caenorhabditis Genetics Center (CGC)
KWN703	*fndc-1(rny14)* II	Lim et al., 2019
KWN638	*rny15 [mRuby3::fndc-1]* II	Lim et al., 2019
NK3282	*pink-1(tm1779)* II; *qy174 [nduv-2::mNG]* V	This study
NK3283	*dct-1(luc184)* X; *qy174 [nduv-2::mNG]* V	This study
NK3284	*fndc-1(rny14)* II; *qy174 [nduv-2::mNG]* V	This study
NK3285	*rny15 [mRuby3::fndc-1]* II; *qy174 [nduv-2::mNG]* V	This study
NK3286	*rny15 [mRuby3::fndc-1]* II; *ojIs23 [pie-1p::SP12::GFP + unc-119(+)]* V	This study
LB138	*him-8(e1489)* IV; *uaDf5/+*	Caenorhabditis Genetics Center (CGC)
NK3287	*rny15 [mRuby3::fndc-1]* II; *uaDf5/+*	This study
NK3288	*fndc-1(rny14)* II; *uadf5/+*	This study
OD70	*unc-119(ed3)* III; *ltIs44 [pie-1p::mCherry::PH(PLC1delta1) + unc-119(+)]* V	Caenorhabditis Genetics Center (CGC)
SHX324	*zju136 [fzo-1::GFP]* II	Caenorhabditis Genetics Center (CGC)

Supplementary Table 2. Oligonucleotides used in the study. Oligonucleotides used for *uaDf5* and total mtDNA level measurements, genotyping strains, and genome editing.

**Oligonucleotides used for *uaDf5* level measurements, genotyping and genome editing**			
**Oligonucleotide sequence (5' → 3')**	**Primer type**	**Amplicon**	**Template**
***uaDf5*:Total mtDNA measurements**			
gtccttgtggaatggttgaatttac	Forward	Wild-type mtDNA (*uaDf5* region)	mtDNA from LB138, NK3288
gtacttaatcacgctacagcagc	Reverse	Wild-type mtDNA (*uaDf5* region)	mtDNA from LB138, NK3289
agcgtcatttattgggaagaagac	Forward	Total mtDNA (*nd1* region)	mtDNA from LB138, NK3290
aagcttgtgctaatcccataaatgt	Reverse	Total mtDNA (*nd1* region)	mtDNA from LB138, NK3291
**Genotyping primers**			
atgtctatgaaacgattcgg	Forward	*pink-1* genotyping	NK3282 genomic DNA
ccgcatctgtacggataa	Reverse	*pink-1* genotyping	NK3282 genomic DNA
gtttcagagacgggtcttt	Forward	*dct-1* genotyping	NK3283 genomic DNA
tttccggcaaacagc	Reverse	*dct-1* genotyping	NK3283 genomic DNA
aatccgtctgatggctgtca	Forward	*fndc-1* genotyping	NK3284, NK3288 genomic DNA
tccacatttaggcgacgctaa	Reverse	*fndc-1* genotyping	NK3284, NK3288 genomic DNA
tcggctactgcatttacttcg	Forward	*tomm-20::mNG* genotyping	NK2747
gagcttcctacaggcttgaa	Reverse	*tomm-20::mNG* genotyping	NK2747
agatgtcgttggcatcgaacgt	Forward	*nduv-2::mNG* genotyping	NK2845
cttgatcggtggtgatagctga	Reverse	*nduv-2::mNG* genotyping	NK2845
**Genome editing**			
gacaccgacgacttggagtaa		*tomm-20* sgRNA sequence	
ggctgctcttaaataaacgct		*nduv-2* sgRNA sequence	

Supplementary Fig. 1: Uncropped confocal fluorescence images from Fig. 3, 4, and 5.

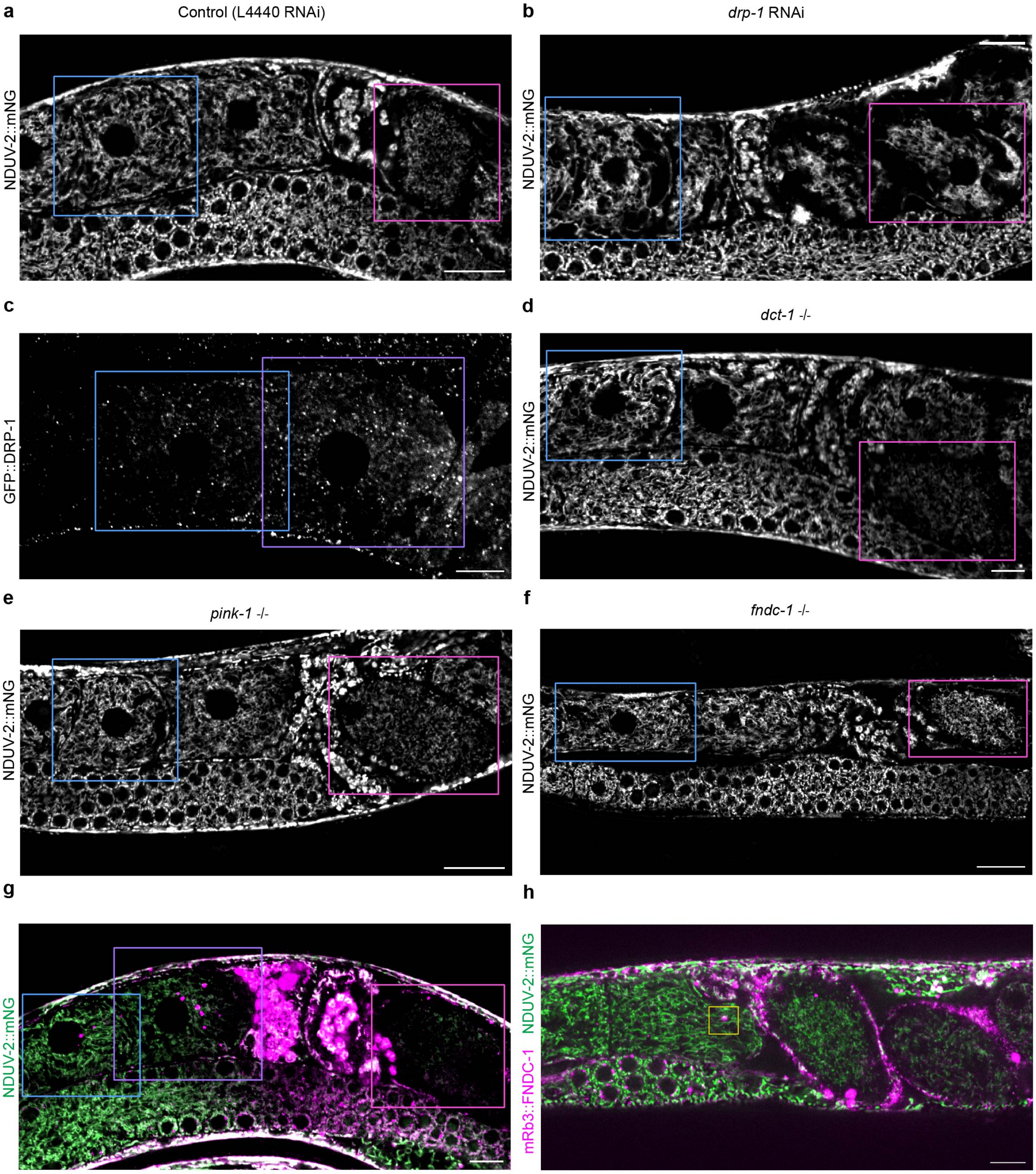

Supplementary Fig. 2: Uncropped confocal fluorescence images from Fig. 2 and Extended Data Fig. 2 and 4.

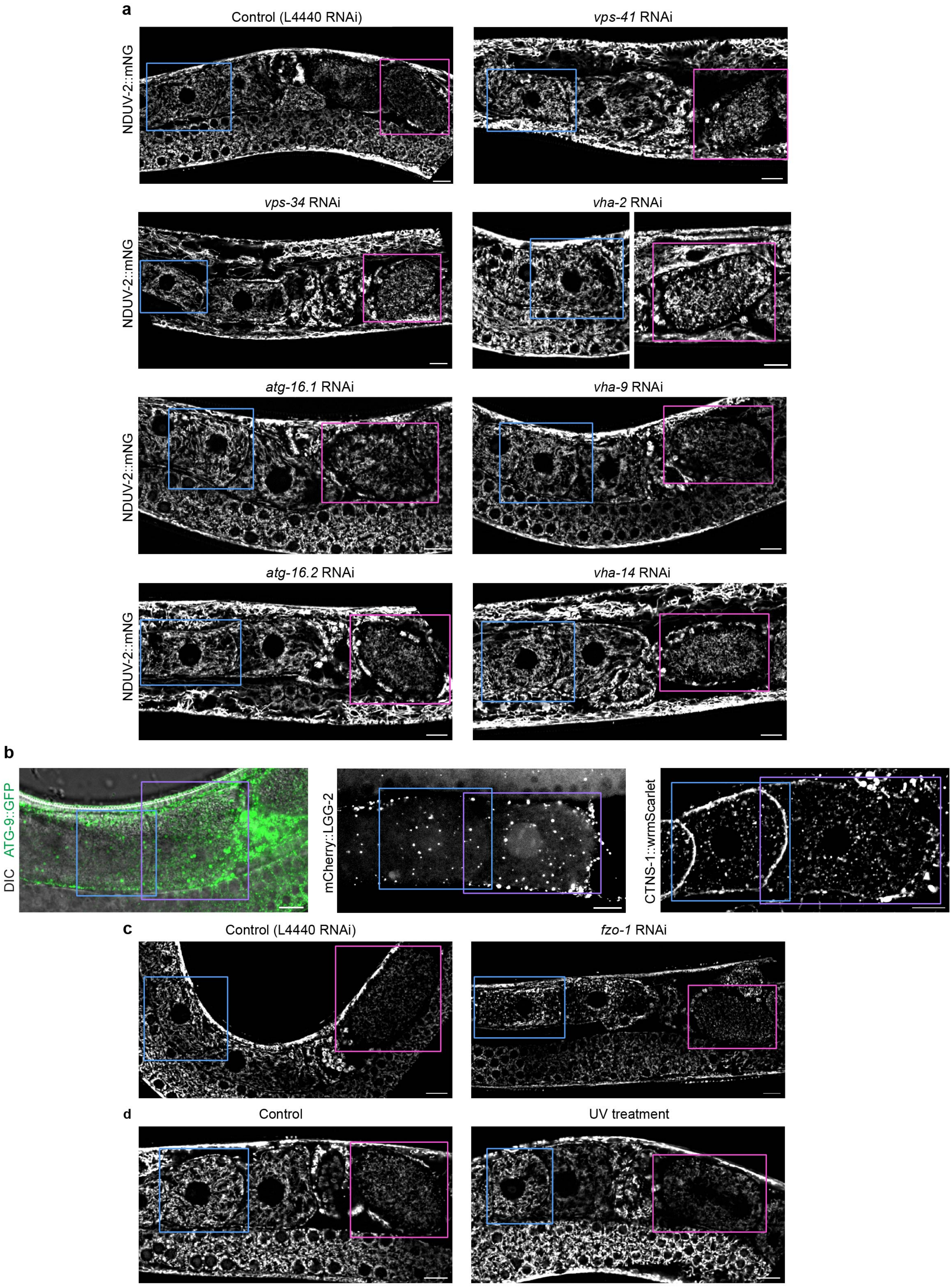

Supplementary Fig.3: Knockdown efficiency of drp-1 and fzo-1 RNAi for experiments in Fig. 3 and Extended Data Fig. 2.

a

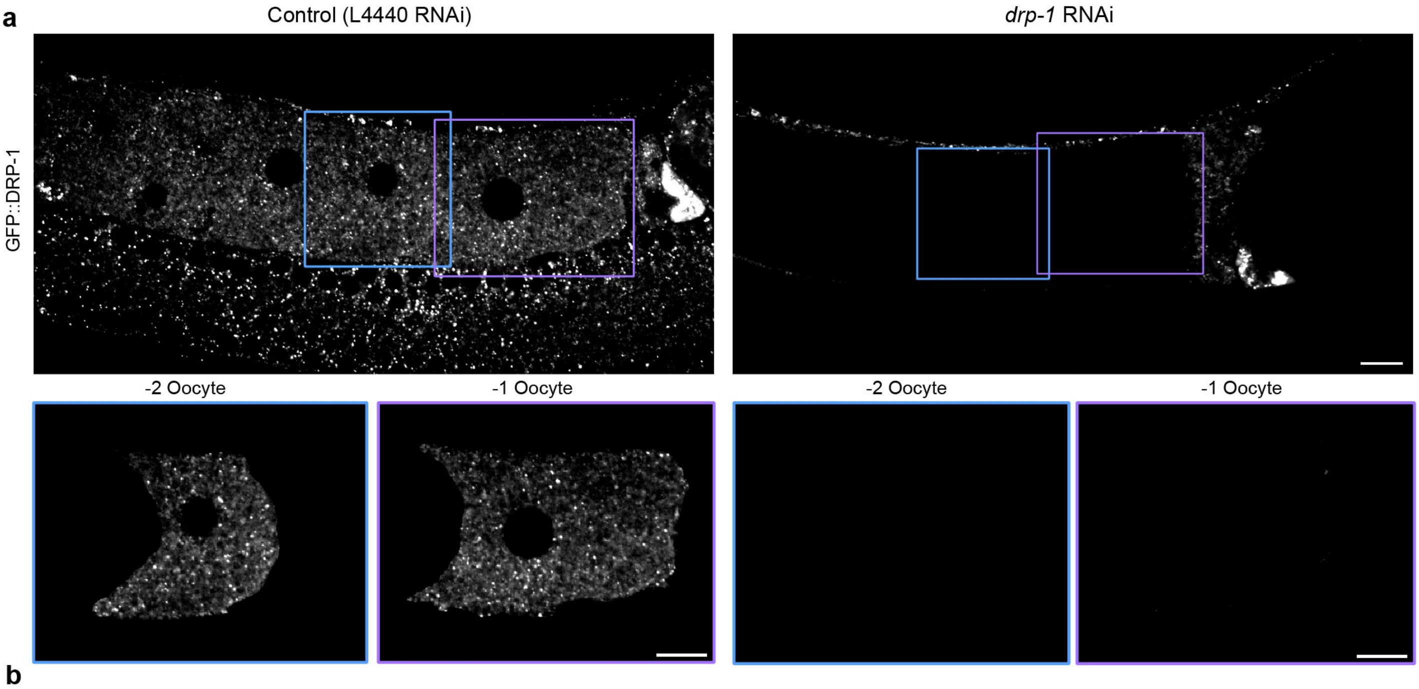

b

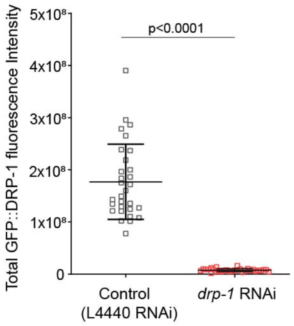

$$\text{Knockdown efficiency \%} = \frac{\text{(Total fluorescence intensity in -2 oocyte of control animal)} - \text{(Total fluorescence intensity in -2 oocyte of } drp\text{-}1 \text{ RNAi treated animal)}}{\text{(Total fluorescence intensity in -2 oocyte of control animal)}} \times 100\ \%$$

Knockdown efficiency of *drp-1* RNAi = 95.65% (n=30 animals)

c

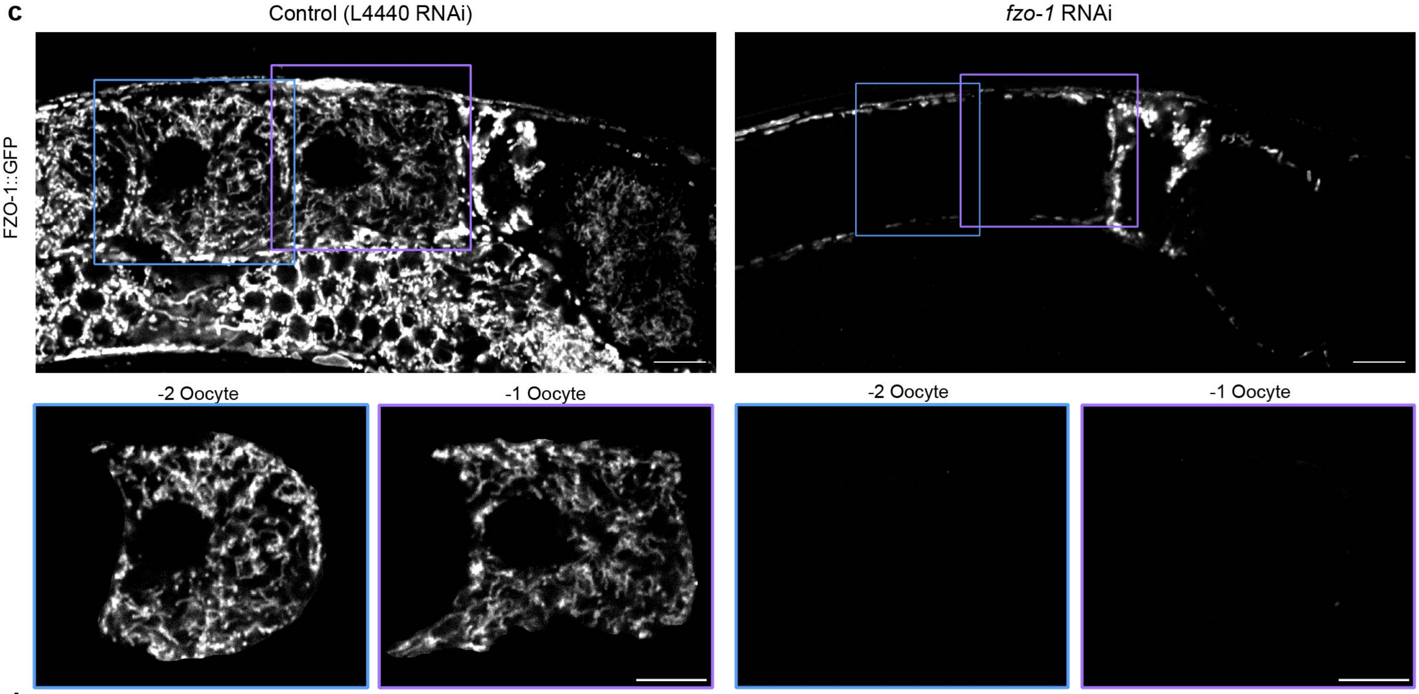

d

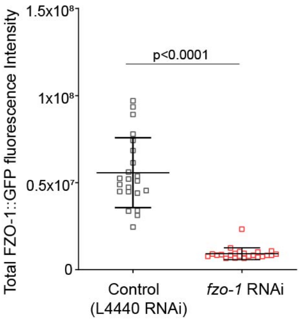

$$\text{Knockdown efficiency \%} = \frac{\text{(Total fluorescence intensity in -2 oocyte of control animal)} - \text{(Total fluorescence intensity in -2 oocyte of } fzo\text{-}1 \text{ RNAi treated animal)}}{\text{(Total fluorescence intensity in -2 oocyte of control animal)}} \times 100\ \%$$

Knockdown efficiency of *fzo-1* RNAi = 84.42% (n=23 animals)

Supplementary Figure legends

Supplementary Fig. 1: Uncropped confocal fluorescence images from main Fig. 3, 4 and 5.
a,**b**, Shown in Fig. 3a. Uncropped fluorescence images showing mitochondria (visualized with NDUV-2::mNG) in the -2 oocyte, -1 oocyte and zygote in an empty vector control (L4440) (**a**) compared to a *drp-1* RNAi-treated animal (**b**).
c, Shown in Fig. 3e. Uncropped fluorescence images showing endogenous GFP::DRP-1 distribution in the -2 oocyte and -1 oocyte.
d,**e**,**f**, Shown in Fig. 4a. Uncropped fluorescence images showing mitochondria (NDUV-2::mNG) in the -2 oocyte, -1 oocyte and zygote in null mutants of *dct-1*(*luc194*) (**d**), *pink-1*(*tm1779*) (**e**), and *fndc-1*(*rny14*) (**f**).
g, Shown in Fig. 5a. Merged uncropped fluorescence images showing mitochondria (NDUV-2::mNG) and mRb3::FNDC-1 in the -2 oocyte, -1 oocyte and zygote.
h, Shown in 5f, Supplementary Video 1. Merged uncropped fluorescence images showing mitochondrial network (NDUV-2::mNG) and mRb3::FNDC-1 punctae in the -2 oocyte, -1 oocyte and zygote. Yellow box indicates the region magnified and tracked for time-lapse imaging shown in Fig. 5f.
Blue boxes indicate -2 oocytes, violet boxes indicate -1 oocytes, and pink boxes indicate zygotes. Scale bars, 10µm.

Supplementary Fig. 2: Uncropped confocal fluorescence images from Main Fig. 2 and Extended Data Fig. 2 and 4.

a, Shown in Fig. 2a. Uncropped fluorescence images showing mitochondria (visualized with NDUV-2::mNG) in the -2 oocyte, -1 oocyte and zygote in an empty vector control (L4440) compared to animals treated with RNAi targeting key macroautophagy genes *vps-34*, *atg-16.1*, *atg-16.2*, *vps-41*, *vha-2*, *vha-9* and *vha-14*.

b, Shown in Fig. 2c,d and e. Left to right: Uncropped fluorescence images showing endogenous ATG-9::GFP, germline specific LC3 (mCherry::LGG-2, *pie-1p::mCherry::lgg-2*) and lysosome (visualized with CTNS-1::wrmScarlet) distribution in the -2 oocyte and -1 oocyte.

c, Shown in Extended Data Fig. 2a. Uncropped fluorescence images showing mitochondria (NDUV-2::mNG) in the -2 oocyte, -1 oocyte and zygote in an empty vector control (L4440) (left) compared to a *fzo-1* RNAi-treated animal (right).

d, Shown in Extended Data Fig. 4d. Uncropped fluorescence images showing mitochondria (NDUV-2::mNG) in the -2 oocyte, -1 oocyte and zygote in control animal (left) compared to a UVC treated animal (right).

Blue boxes indicate -2 oocytes, violet boxes indicate -1 oocytes and pink boxes indicate zygotes. Scale bars, 10µm.

Supplementary Fig. 3: Knockdown efficiency of *drp-1* and *fzo-1* RNAi for experiments in Fig. 3 and Extended Data Fig. 2.

a, Endogenous GFP::DRP-1 puncta distribution in the germline of an adult empty vector control (L4440) (left) compared to a *drp-1* RNAi-treated animal (right). Blue boxes indicate -2 oocytes and violet boxes -1 oocytes. -2 oocytes and -1 oocytes enclosed in boxed regions are magnified. Scale bars, 10µm.

b, Left: Total GFP::DRP-1 fluorescence intensity in -2 oocytes compared between empty vector controls (L4440) and *drp-1* RNAi-treated animals. Data represent mean ± s.d. (n = 30 animals per condition) from three biological replicates. P value using two-tailed Student's *t*-test. Right: Knockdown efficiency calculation for *drp-1* RNAi.

c, Endogenous FZO-1::GFP (*fzo-1::gfp*) distribution in the germline of an adult empty vector control (L4440) (left) compared to a *fzo-1* RNAi-treated animal (right). Blue boxes indicate -2 oocytes, and violet boxes indicate -1 oocytes. -2 oocytes and -1 oocytes enclosed in boxed regions are magnified. Scale bars, 10μm.

d, Left: Total FZO-1::GFP fluorescence intensity in -2 oocytes compared between empty vector controls (L4440) and *fzo-1* RNAi-treated animals. Data represent mean ± s.d. (n = 23 animals per condition) from three biological replicates. P value using Mann-Whitney test. Right: Knockdown efficiency calculation for *fzo-1* RNAi.

Supplementary Video 1

Mitochondrial fragmentation events during *C. elegans* OZT; FUNDC1 puncta mark sites of mitochondrial fragmentation.

Supplementary Video 2

Dynamic interactions of FUNDC1 puncta with -1 oocyte mitochondria.

Supplementary Files

This is a list of supplementary files associated with this preprint. Click to download.

- SuppVid2copy.mp4
- SuppVid1mp4copy.mp4
